# A PEDF-Derived Peptide Inhibits Retinal Neovascularization and Blocks Mobilization of Bone Marrow-Derived Endothelial Progenitor Cells

**DOI:** 10.1155/2012/518426

**Published:** 2011-06-28

**Authors:** Richard Longeras, Krysten Farjo, Michael Ihnat, Jian-Xing Ma

**Affiliations:** ^1^Department of Physiology, University of Oklahoma Health Sciences Center, Oklahoma, OK 73104, USA; ^2^Department of Cell Biology, University of Oklahoma Health Sciences Center, Oklahoma, OK 73104, USA

## Abstract

Proliferative diabetic retinopathy is characterized by pathological retinal neovascularization, mediated by both angiogenesis (involving mature endothelial cells) and vasculogenesis (involving bone marrow-derived circulating endothelial progenitor cells (EPCs)). Pigment epithelium-derived factor (PEDF) contains an N-terminal 34-amino acid peptide (PEDF-34) that has antiangiogenic properties. Herein, we present a novel finding that PEDF-34 also possesses antivasculogenic activity. In the oxygen-induced retinopathy (OIR) model using transgenic mice that have Tie2 promoter-driven GFP expression, we quantified Tie2GFP^+^ cells in bone marrow and peripheral blood by fluorescence-activated cell sorting (FACS). OIR significantly increased the number of circulating Tie2-GFP^+^ at P16, correlating with the peak progression of neovascularization. Daily intraperitoneal injections of PEDF-34 into OIR mice decreased the number of Tie2-GFP^+^ cells in the circulation at P16 by 65% but did not affect the number of Tie2-GFP^+^ cells in the bone marrow. These studies suggest that PEDF-34 attenuates EPC mobilization from the bone marrow into the blood circulation during retinal neovascularization.

## 1. Introduction

Vascular development is regulated by a tight and complex balance between pro- and anti-angiogenic factors such as vascular endothelial growth factor (VEGF) and pigment epithelium-derived factor (PEDF), respectively [1–4]. This balance is important to maintain homeostasis of blood vessel formation. Under certain pathological conditions, such as proliferative diabetic retinopathy, age-related macular degeneration, tumorigenesis, and rheumatoid arthritis, this balance is disrupted, leading to pathological neovascularization [1, 3]. Pathological neovascularization involves two distinct mechanisms, vasculogenesis, in which bone marrow-derived endothelial progenitor cells (EPCs) contribute to new blood vessel formation [5–8], and angiogenesis, in which existing mature endothelial cells proliferate and migrate to form new blood vessels [9, 10]. Several angiogenic inhibitors have been identified which effectively inhibit pathological neovascularization, but the effect of such antiangiogenic factors on vasculogenesis has not been established. 

PEDF is a 50-kDa secreted glycoprotein bearing multiple biological activities [11–15], including potent anti-angiogenic activity, which has been shown to inhibit pathological neovascularization, such as retinal neovascularization, which occurs during proliferative diabetic retinopathy [12, 14, 15]. However, the effect of PEDF on vasculogenesis has not been documented. Recently, a 34-amino acid peptide derived from the N-terminus of PEDF (PEDF-34) was found to possess intact *ex vivo* antiangiogenic activity and inhibit choroidal neovascularization in rats [16, 17]. In the present study, we investigated the direct effects of PEDF-34 on the proliferation and viability of primary endothelial cell cultures and on *in vivo* blood vessel development using the chicken chorioallantoic membrane (CAM) assay. We also used the oxygen-induced retinopathy (OIR) mouse model to assess the ability of the PEDF-34 to inhibit retinal neovascularization. Using transgenic mice that express GFP under the control of the endothelial cell-lineage specific promoter Tie2 (Tie2-GFP mice), we quantified bone marrow-derived EPCs and circulating endothelial cells by fluorescence-activated cell sorting (FACS) of Tie2-GFP^+^ cells. These studies are the first to demonstrate that systemic administration of PEDF-34 peptide is sufficient to inhibit retinal neovascularization. We also demonstrate for the first time that retinal neovascularization in the OIR model coincides with a spike in the number of circulating endothelial cells and EPCs. Furthermore, PEDF-34 blocks the spike in circulating endothelial cells and EPCs during OIR. These data suggest that in addition to its localized anti-angiogenic effects on neovascular lesions, PEDF may also have a systemic activity that blocks the release of EPCs from bone marrow to reduce EPC-mediated vasculogenesis during retinal neovascularization.

## 2. Materials and Methods

### 2.1. PEDF-34 Peptide

The PEDF-34 peptide, spanning from amino acids 44 to 77 of the N-terminus of the PEDF protein, was chemically synthesized by Proteintech lab (Chicago, IL) and purified by HPLC. Proper synthesis of the peptide was controlled by mass spectrometry.

### 2.2. Bovine Retinal Capillary Endothelial Cell (BRCEC) Isolation and Culture

BRCEC were isolated from whole retinas of cows younger than 18 months in accordance with USDA regulations. Briefly, retinas were carefully singled out from eyecups, washed, homogenized, and digested prior to being applied to a series of filters. Cells were grown in collagen-coated flask in the presence of 10% human serum in Dulbecco's modified eagle medium (DMEM) containing low glucose (1 g/L) until confluency. The endothelial cell identity of the BRCEC cultures was confirmed by their ability to uptake acetylated low-density lipoprotein (LDL) labeled with 1,1′-dioctadecyl-3,3,3′,3′-tetramethylindocarbocyanine (Dil-Ac-LDL) (Harbor Bio-Products). Following a 1 hr incubation with Dil-Ac-LDL, cells were fixed in 4% paraformaldehyde, counterstained with DAPI included in mounting media (Vectorlabs, Burlingame), and examined by fluorescence microscopy. Only BRCEC preparations with more than 95% purity were used in this study.

### 2.3. Cell Viability Assays

A cell line derived from rat Müller cells, rMC-1, was kindly provided by Dr. Sarthy at Northwestern University. BRCECs between passages 3 and 8 were seeded in gelatin-coated 48 well plates. BRCECs and rMC-1 cells were maintained in regular DMEM growth media until the assay. Then, the growth media were replaced with DMEM containing 1% FBS, low glucose (1 g/L), and 1% antibiotic/antimycotic, and the cells were treated with PEDF-34 in various concentrations for 72 h. At the end of the treatment, an MTT assay (Roche, Nonnenwald Germany) was performed according to the manufacturer's recommendation. Data were collected on a Victor plate reader.

### 2.4. Detection of Apoptosis by FACS Analysis

Adherent BRCECs were trypsinized for 2 min, and the trypsinization was stopped with PBS/10% fetal calf serum. Then cells were washed twice with annexin-binding buffer and incubated with 2.5 *μ*L annexin-PE and 2.5 *μ*L 7AAD for 20 min according to the instructions of the manufacturer (Pharmingen, Annexin V-Pe Apoptosis Kit). The cells were analyzed by fluorescence-activated cell sorting (FACS) using a FACSCalibur flow cytometer and Cell Quest software (BD Biosciences, Rockville, MD).

### 2.5. Chicken Chorioallantoic Membrane (CAM) Assays

CAM assays were used to assess the antiangiogenic potential of PEDF-34 *in vivo*. Briefly, fertilized eggs were incubated at 37°C and 65% relative humidity for three days with a rocking mechanism. The embryos were then removed from the incubator, washed with a 1 : 100 solution of benzalkonium chloride (Research Chemicals, Heyshan Lanes, UK) in distilled water, and cracked out into 100-mm Petri dishes (BD Falcon, Franklin Lakes, NJ). A circular section (1.2 mm diameter) of nitrocellulose was saturated with either PEDF-34 or control peptide at the required concentration. An identical nitrocellulose section was saturated with sterile PBS and used as internal control in each CAM. The nitrocellulose disks were placed onto the surface of the embryo in an area of active vascularization. Each peptide and the control PBS were reapplied onto the disks every 24 h. After 72 h incubation, the disks were carefully removed from the surface of the embryo. Images were taken of the area in the vicinity of the removed disks. Blood vessel density was evaluated by densitometric analysis of the images of the disk areas using ImageJ (NIH).

### 2.6. Induction of Retinal Neovascularization in Mice and Quantification of Retinal Nuclei

Tie2-GFP mice, transgenic mice that express GFP under the control of the Tie-2 promoter were a kind gift from Dr. Sanai Sato. The Tie2-GFP mice at postnatal day 7 (P7) were exposed to hyperoxia (75% O_2_) for 5 days. They were brought back to normoxic room air at P12 and were thereafter maintained at normoxia to induce retinal neovascularization. At P12 the OIR mice were separated into 2 groups: one group was injected intraperitoneally with PEDF-34, once a day from P12 to P17, and the other group was injected with BSA in a similar fashion. At P18, the eyes were enucleated, fixed in 10% paraformaldehyde, and embedded in paraffin. Sagittal sections of 5 *μ*m thickness were made using a microtome (Microm HM 325). Noncontinuous sections were mounted on slides and stained with hematoxylin and eosin as described by Smith et al. [18]. Light microscopy was used to count nuclei of vascular cells present on the vitreal side of the retina. A total of 8 sagittal sections from each eye were examined, and cell numbers were averaged for each group. The average number of preretinal nuclei was compared to the control group by Student's *t*-test.

### 2.7. EPC Isolation

Tie2-GFP mice in FVB background were used in this study. For bone marrow cell isolation, tibias were collected, extensively flushed with PBS and crushed. Bone fragments and cells in suspension were applied to a 100 *μ*M filer and washed with 30 mL of ice-cold PBS. Cells which passed through the filter were centrifuged for 10 min at 1,500 rpm, and the supernatant was discarded. Cells were resuspended in 4% paraformaldehyde and placed on ice shielded from the light. For peripheral blood cell isolation, blood was collected by cardiac puncture of the right ventricle, transferred to a tube with heparin salt, and placed on ice. The blood was then layered onto a histopaque 1083 (Sigma, Saint Louis, MO) and centrifuged for 30 min at 3000 rpm. The mononuclear cell fraction was collected and rinsed with ice-cold PBS. Cells were resuspended in 4% paraformaldehyde and placed on ice shielded from the light.

### 2.8. FACS Analysis of Tie2-GFP^+^ Cells

Cell were fixed for 1 h in 4% paraformaldehyde, and then washed three times in cold PBS. The fixed cells were applied to a FACSCalibur flow cytometer and analyzed using Cell Quest software (BD Biosciences) using a 530 nm filter. In each sample, 1,000,000 events and 30,000 events were counted for bone marrow and circulating mononucleated cells, respectively. Collected data was analyzed by FlowJo software (Tree Star, Ashland, OR, USA).

### 2.9. Immunostaining and Confocal Microscopy of Tie2-GFP^+^ Cells

Tie2-GFP^+^ sorted cells were washed in PBS and adhered to charged histology slides for immunostaining. Cells were preincubated with the appropriate antibody isotype to prevent nonspecific binding of the primary antibody. We then used the following antibodies: anti-CD117 labeled with allophycocyanin (APC) (BD Pharmingen) and rat antimouse CD133 (Chemicon). For CD133, we used an antirat IgG secondary antibody labeled with Alexa 648 (BD Pharmigen). All slides were mounted in Vectorlabs-DAPI. The cells were imaged and analyzed on a Nikon TE2000-E. 

## 3. Results

### 3.1. PEDF-34 Inhibits Cell Proliferation and Induces Apoptosis in BRCEC

**Figure 1:**
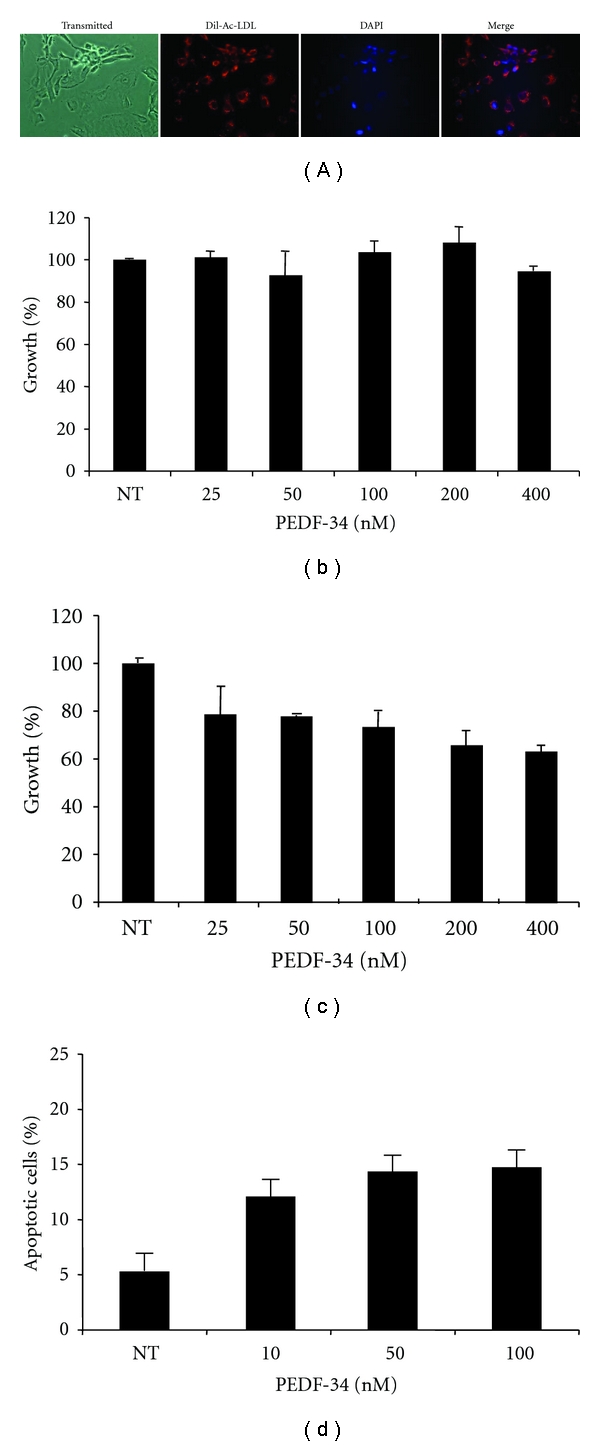
PEDF-34 acts in a concentration-dependent manner to inhibit cell proliferation and induce apoptosis specifically in endothelial cells. (a) The purity of primary BRCEC was examined using Dil-Ac-LDL uptake assays. The cells were counter-stained with DAPI, and exhibited a >99% purity based on positive Dil-Ac-LDL uptake. Both rat Müller cells (b) and BRCEC (c) were treated with increasing concentrations of PEDF 34-mer ranging from 25 to 400 nM. Viable cells were quantified after 72 h by MTT assay and expressed as % of the cells in control treated with the BSA only (denoted as NT on graph). (d) BRCECs were treated with increasing concentrations of PEDF 34-mer for 24 h. Apoptotic cells were quantified by counting Annexin V positive cells using FACS and expressed as % in total cells (mean + SD, *n* = 3).

Full-length PEDF protein is known to target endothelial cells to inhibit proliferation and induce apoptosis [12], and PEDF-34 has previously been shown to inhibit blood vessel sprouting *ex vivo *and inhibit the progression of laser-induced choroidal neovascularization in rats [16]. To evaluate the direct and endothelial cell-specific effects of the PEDF-34 on cell proliferation and viability, both BRCEC and rMC-1 cells were treated with increasing concentrations of PEDF-34. Beforehand, the identity and purity of primary BRCEC cultures were confirmed by evaluating cellular uptake of fluorescently-labeled acetylated LDL (Dil-Ac-LDL), which is exclusively taken up by endothelial cells which express the LDL receptor. More than 99% of the isolated BRCEC were positive for Dil-Ac-LDL uptake (Figure 1(a)). MTT cell proliferation assays showed that rMC-1 cell proliferation was unaffected by up to 400 nM of PEDF-34 (Figure 1(b)). In contrast, the PEDF-34 inhibited BRCEC proliferation in a concentration-dependent manner, with as low as 50 nM causing a 20% decrease in cell proliferation and with 400 nM reducing cell proliferation by more than 30% (Figure 1(c)). These data show that PEDF-34 selectively inhibits cell proliferation in endothelial cells.

To determine whether the PEDF-34 induces apoptosis in BRCEC, Annexin V staining and subsequent FACS analysis was used to quantify apoptotic cells. PEDF-34 increased the percentage of apoptotic cells in a concentration-dependent manner (Figure 1(d)), providing the first evidence of the direct and specific proapoptotic activity of the PEDF-34 in primary endothelial cells.

### 3.2. Locally Delivered PEDF-34 Inhibits New Blood Vessel Formation *In Vivo*

**Figure 2:**
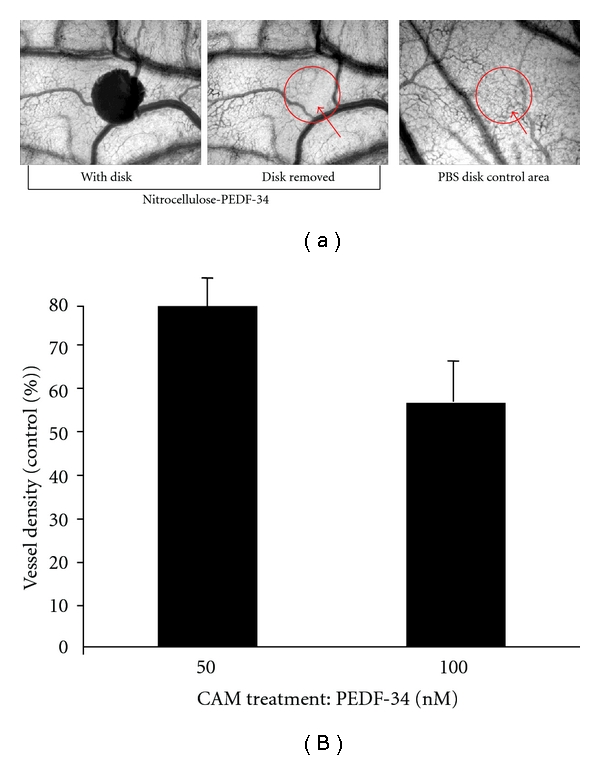
Antiangiogenic activity of PEDF 34-mer during *in vivo* blood vessel formation. CAMs were separately treated with either 50 or 100 nM PEDF-34. (a) Left: close-up image of a chicken embryo CAM with a nitrocellulose disk containing PEDF-34 (100 nM). Middle: the disk area after removal of PEDF-34 nitrocellulose disk. Right: image of a PBS-treated-nitrocellulose control area on the same CAM with the disk removed. Note considerable reduction in the overall number of small newly formed blood vessels, when compared with the PBS-treated control. (b) Blood vessel density in the CAMs was assessed by software analysis. Vascular densities in the disk areas of CAMs treated with 50 and 100 nM were quantified and averaged. The graph represents the percent of vascular density found under PEDF-34-treated areas compared to the PBS-treated control area, which was set at 100%. Vascular density in the CAM treated with PEDF-34 is significantly lower than the control (mean + SD, *n* = 5).

The chorioallantoic membrane (CAM) of a growing chicken egg was used as a model system to test the effects of PEDF-34 on *in vivo* blood vessel formation [19]. CAMs were treated with small disks of nitrocellulose saturated with PEDF-34. Nitrocellulose saturated with PBS alone was included in every CAM as an internal negative control. After 72 h of treatment, the areas of CAMs covered by disks containing PEDF-34 showed fewer blood vessels compared to the areas covered by disks containing PBS alone (Figure 2(a)). The vessel density in the area covered by the disk was quantified by computer analysis of digital images and averaged. The results showed that 50 nM of PEDF-34 decreased vessel density by up to 20%, and 100 nM PEDF-34 decreased vessel density by 45%, compared to the average vascular density of the control (Figure 2(b)), suggesting a dose-dependent inhibitory effect of PEDF-34 on *in vivo* blood vessel formation.

### 3.3. Systemic Administration of PEDF-34 Inhibits Pathological Retinal Neovascularization in the OIR Model

**Figure 3:**
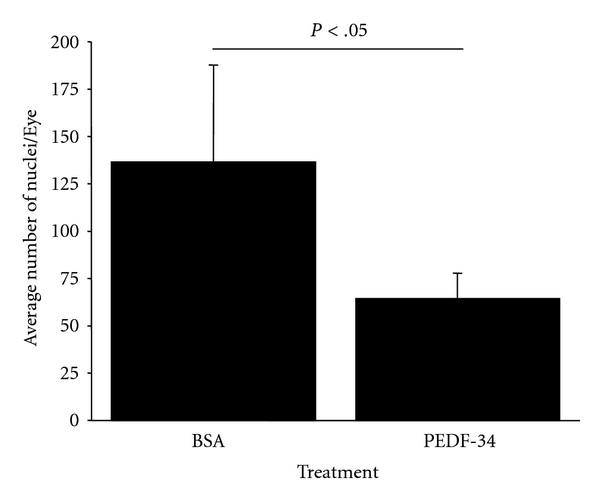
Systemic injection of PEDF-34 reduced ischemia-induced retinal neovascularization. The graph represents the average number of preretinal (vitreous) vascular cells in OIR mice treated with BSA or PEDF-34. (mean ± SEM, BSA *n* = 4. PEDF-34 *n* = 5).

To evaluate the effects of PEDF-34 on vasculogenesis during pathogenic neovascularization, we used the OIR mouse model, which initiates the pathogenesis of retinal neovascularization beginning at P12 [18]. OIR mice received daily intraperitoneal injections of either PEDF-34 (5 mg/kg of body weight) or the same amount of BSA as control from age P12 to P17. At P18, neovascularization was quantified by counting pre-retinal nuclei in 8 discontinuous sections per eye (Figure 3). The mean preretinal neovascular cell number in the BSA treated group was 136 ± 50 (mean ± SD, *n* = 4) per section. Systemic treatment with PEDF-34 reduced the number of preretinal neovascular cells by approximately 50% to only 64 ± 13 (mean ± SD, *n* = 5) per section, significantly lower than that in the BSA-treated group (*P* < .05, *n* = 5) (Figure 3). These results demonstrate that systemic injection of PEDF-34 prevented the progression of ischemia-induced retinal neovascularization.

### 3.4. Isolation and Characterization of Tie2-GFP^+^ Cells

**Figure 4:**
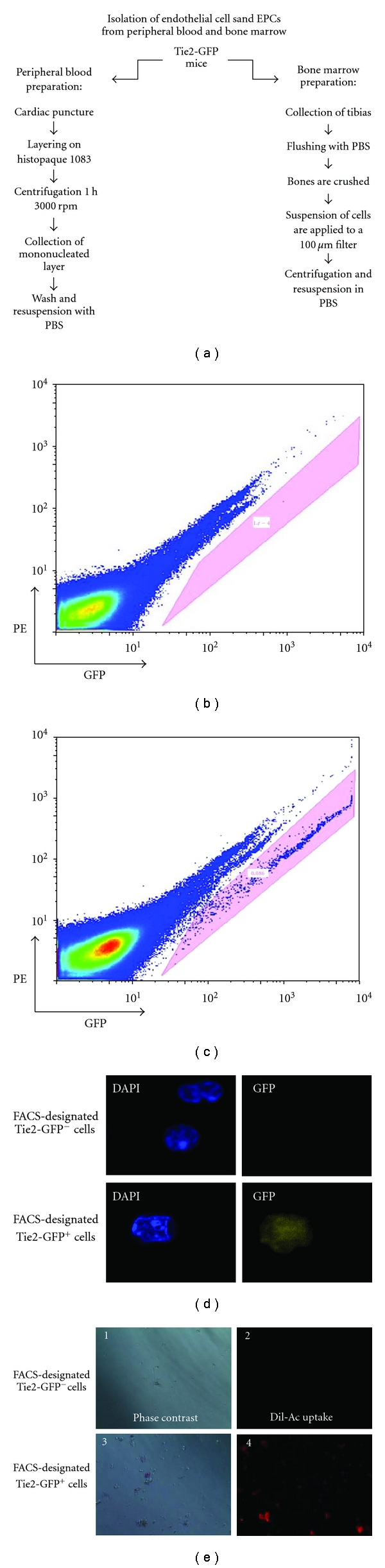
Isolation of Tie2-GFP^+^ cells from bone marrow and peripheral blood in mice. (a) Isolation of bone marrow and peripheral blood cells was performed in parallel as depicted. Washed cells were resuspended in ice-cold PBS and shielded from the light to prevent photobleaching. (b) and (c) Flow cytometric analysis of isolated cells. Resuspended cells were subjected to FACS on an Influx cell sorter. The profile obtained from Tie2-GFP mice (c) exhibited an additional population when compared to the wild-type C57BL/6 (b) (denoted in the shaded pink box). Therefore, this exclusive population was characteristic of the Tie2-GFP^+^ cells. (d) Tie2-GFP^−^ and Tie2-GFP^+^ cells as identified by FACS analysis were collected separately, fixed onto slides, counter-stained with DAPI, and visualized by fluorescence microscopy. Only cells identified by prior FACS analysis as Tie2-GFP^+^ exhibited significant levels of green fluorescence by microscopic (100x) analysis. (e) Tie2-GFP^+^ cells which were isolated by FACS from Tie2-GFP mice were viable and of the endothelial cell lineage, as demonstrated by their ability to uptake Dil-Ac-LDL. Cells sorted by FACS as Tie2-GFP^−^ did not have the ability to uptake Dil-Ac-LDL.

In order to study circulating endothelial cells during OIR, we used Tie2-GFP transgenic mice, which have GFP expression exclusively in cells of endothelial cell lineage, including mature endothelial cells and bone marrow-derived endothelial progenitor cells. Prior to commencing OIR studies, we performed preliminary analyses to detect and characterize Tie2-GFP^+^ cells from bone marrow and peripheral blood. Bone marrow cells and peripheral blood mononuclear cells were harvested separately as described in Section 2 and depicted in Figure 4(a), and then placed on ice and shielded from direct light. Cells were then fixed in 4% paraformaldehyde and subjected to FACS to sort Tie2-GFP^+^ cells. Bone marrow cells collected from wild type C57bl/6 mice were used as a negative control. Cells collected from Tie2-GFP mice exhibited a distinctly shifted population of Tie2-GFP^+^ cells that was absent in cells isolated from wild-type mice (Figures 4(b) and 4(c)). The efficacy of FACS-mediated separation of Tie2-GFP^−^ and Tie2-GFP^+^ cells was evaluated by collecting the FACS-separated cell populations and performing postanalysis using fluorescence microscopy. After staining both cell populations with DAPI, GFP fluorescence is only visible in the population of cells identified as Tie2GFP^+^ by FACS analysis (Figure 4(d)). Thus, FACS analysis is a reliable method for isolating and quantifying Tie2GFP^+^ cells. 

To confirm the endothelial identity of FACS-isolated Tie2-GFP^+^ cells, cell populations designated as Tie2-GFP^−^ and Tie2-GFP^+^ cells were placed separately into 96-well plates for Dil-Ac-LDL uptake assays. As expected, Dil-Ac-LDL uptake only occurred in the designated Tie2-GFP^+^ cells (Figure 4(e)). This confirmed the endothelial identity of Tie2-GFP^+^ cells and also the reliability of the FACS-based isolation and quantification methods. 

**Figure 5:**
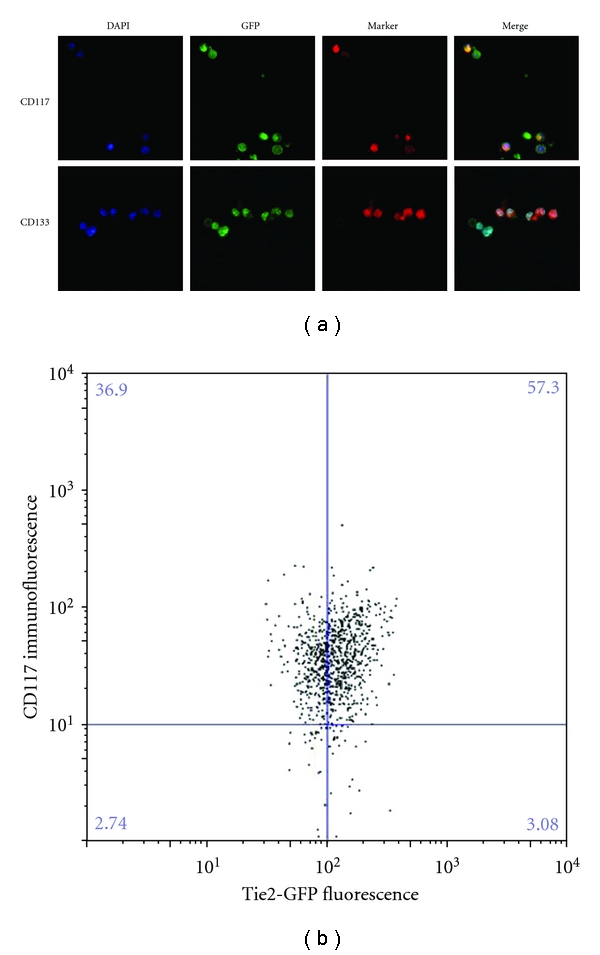
Characterization of circulating Tie2-GFP^+^ cells as EPCs. (a) Tie2-GFP^+^ cells from Tie2-GFP mice were sorted onto slides and immunostained for the EPC-specific markers CD117 and CD133 and counterstained with DAPI. Confocal microscopy was used to capture images at 40x magnification; (b) EPCs were fixed, sorted for GFP, then immunostained for CD117, and subjected to FACS. Most Tie2-GFP^+^ cells stained positive for CD117, indicating the majority of circulating endothelial cells are EPCs.

To confirm that the Tie2-GFP^+^ cells represent EPCs in addition to circulating mature endothelial cells, the Tie2-GFP^+^ cells were immunostained for CD117 and CD133, two established markers of EPCs [20–23]. CD117 and CD133 immunostaining of Tie2-GFP^+^ cells was easily observed (Figure 5(a)), and quantification by FACS revealed that at least 90% of sorted Tie2-GFP^+^ cells exhibited significant staining for CD117 (Figure 5(b)). These results show that a significant portion of the Tie2-GFP^+^ cells are EPCs.

### 3.5. The Number of Circulating Tie2-GFP^+^ Cells Correlates with the Progression of Retinal Neovascularization in the OIR Model

**Figure 6:**
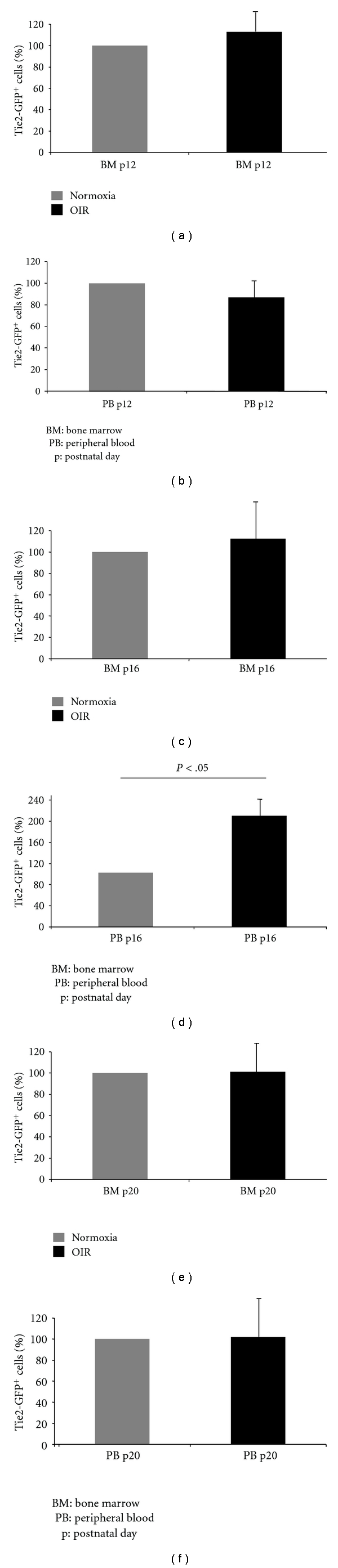
The number of circulating endothelial cells and EPCs increases in OIR mice at P16. Tie2-GFP^+^ cells in both the bone marrow (BM) and peripheral blood (PB) from Tie2-GFP mice were quantified by FACS at p12, p16 and p20 under normoxic rearing conditions (grey), or in the OIR model (black). Graphs represent the percent of Tie2-GFP^+^ cells based on 100% being set to the average number of Tie2-GFP^+^ cells from normoxic mice.

In order to characterize the correlation between circulating endothelial cells (including both EPCs and mature endothelial cells) and retinal neovascularization in the OIR model, Tie-2-GFP mice were exposed to 75% oxygen from age P7 to P12 and then returned to room air to induce retinal neovascularization [18]. Tie2-GFP^+^ cells from both bone marrow and peripheral blood were quantified by FACS. Age-matched Tie2-GFP mice maintained in constant room air were used as non-OIR controls. In bone marrow, there was no significant increase in Tie2-GFP^+^ cells at P12, P16 and P20, compared to age-matched non-OIR controls (Figures 6(a), 6(c), and 6(e)) suggesting the oxygen treatment does not influence the percentage of Tie2-GFP^+^ cells present in the bone marrow. In contrast, in peripheral blood, there was a significant increase in the number of circulating Tie2-GFP^+^ cells at age P16, but not at P12 and P20, when compared to age-matched controls (Figures 6(b), 6(d), and 6(f)). P16 correlates with the most aggressive stage of retinal neovascularization which occurs from P16–P18 [18]. Thus, the peak in the number of circulating endothelial cells coincides with the peak of retinal neovascularization, which strongly suggests circulating endothelial cells contribute to the pathogenesis of retinal neovascularization.

### 3.6. PEDF-34 Blocks the Increase in Circulating Tie2-GFP^+^ Cells during Retinal Neovacularization in the OIR Model

**Figure 7:**
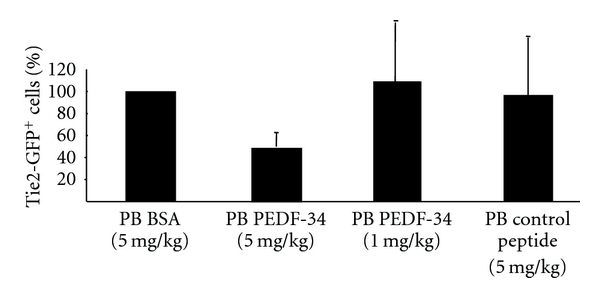
PEDF-34 reduces the number of circulating endothelial cells and EPCs in OIR mice. In the OIR model, Tie2-GFP pups received a daily i.p. injection of either PEDF-34, BSA, or a control peptide in PBS at a dose of 5 mg/kg of body weight from P12 to P16. At P16, Tie2-GFP^+^ circulating endothelial cells and EPCs in peripheral blood were quantified by FACS. The graph represents the percentage of Tie2-GFP^+^ cells in peripheral blood, based on 100% being set to the average number of Tie2-GFP^+^ cells from OIR mice injected with BSA alone.

To evaluate the potential for PEDF-34 to act on circulating endothelial cells, OIR was induced in Tie2-GFP mice, and once mice were returned to normoxic room air at P12 to induce retinal neovascularization, mice received daily i.p. injections of PEDF-34 from P12 up to P17. Control OIR mice received an equivalent quantity of BSA. Tie2-GFP^+^ cells from peripheral blood and bone marrow were quantified by FACS at P16. The injection of PEDF-34 (5 mg/kg body weight) resulted in a 55% reduction in the number of circulating Tie2-GFP^+^ cells in the peripheral blood (Figure 7), but did not affect the number of Tie2-GFP^+^ cells in the bone marrow (see Supplemental Figure  1 in Supplementary Material available online at doi: 10.1155/2012/518426). A lower dose of PEDF-34 (1 mg/kg body weight) did not result in a significant decrease in the number of circulating Tie2-GFP^+^ cells (Figure 7). These results suggest that PEDF-34-mediated inhibition of Tie2-GFP^+^ cells in circulation is both highly specific and dose-dependent.

### 3.7. The Effect of PEDF-34 on Circulating Tie2-GFP^+^ Cells Is Not via Regulation of VEGF

The regulation of EPC release from the bone marrow is not well understood. However, VEGF has previously been shown to enhance pathological neovascularization partially by increasing the release of EPCs from bone marrow into the blood circulation [6]. Furthermore, we have previously demonstrated that PEDF competes with VEGF for binding to VEGF receptor 2 on endothelial cells [24], which is suggested to be a mechanism for the antiangiogenic activity of PEDF. Thus, to determine if PEDF-34 reduces circulating endothelial cells during retinal neovascularization by targeting VEGF-mediated EPC release from bone marrow, we tested the effect of PEDF-34 on plasma VEGF levels. Tie2-GFP mice with OIR received daily i.p. injections of PEDF-34 or BSA (5 mg/kg body weight) from P12 to P15. VEGF concentrations in the plasma were measured by ELISA at P16. PEDF-34 did not affect VEGF plasma levels, compared to the group treated with BSA (Supplemental Figure  2).

## 4. Discussion

Pathological neovascularization is a common cause of vision loss in diabetic retinopathy. With the number of patients affected by diabetes growing rapidly, it has become a major public health quest to arrest and prevent neovascular complications associated with diabetes. Although several anti-VEGF therapies have displayed beneficial effects for the treatment of diabetic retinopathy, patients often become refractive to anti-VEGF therapy. Thus, more drugs with different molecular mechanisms need to be developed. Vasculogenesis was previously considered to occur primarily during physiological development of vasculature, but recent evidence indicates that EPC-mediated vasculogenesis also contributes to pathological neovascularization, including retinal neovascularization [4, 5, 7, 20]. Thus, blockade of the release of EPCs from the bone marrow into the circulation represents a new target for pharmacological intervention of pathological neovascularization. 

Endogenous antiangiogenic proteins have been intensively studied over the past few years due to their therapeutic potential in the treatment of neovascular disorders, including proliferative diabetic retinopathy [25]. Their regulatory roles in angiogenesis have been well established *in vitro* and *in vivo* [12, 15, 26, 27], although their effects on circulating endothelial cells and EPC-mediated vasculogenesis have not been investigated previously. The data presented herein demonstrates that a 34-amino acid peptide fragment derived from the N-terminus of the angiogenic inhibitor PEDF inhibits circulating endothelial cells and EPCs during the pathogenesis of retinal neovascularization in the OIR model. 

The OIR model is commonly used to study retinal neovascularization [18], because it has a well-characterized and highly reproducible course of retinal neovascularization. In the OIR model, transient exposure to hyperoxia from P7 to P11, followed by return to normoxia at P12 causes ischemia-induced retinal neovascularization that involves increased VEGF expression, decreased PEDF expression, and increased vascular leakage [3, 15]. Previous studies have shown that these features of retinal neovascularization are transient and peak at P16–P18. The present study is the first to demonstrate that the number of circulating endothelial cells and EPCs also peaks at P16 during OIR, correlating with the peak in VEGF expression and the most aggressive stage of retinal neovascularization. This correlation provides strong evidence that the increase in circulating endothelial cells and EPCs contributes to the retinal neovascularization in the OIR model. In contrast, EPC abundance in bone marrow is not changed in OIR mice at any of the time points analyzed. However, it is likely that bone marrow-derived EPCs are mobilized and contribute to the spike in circulating endothelial cells and EPCs, and that their numbers are so quickly replenished in the bone marrow that no mobilization-induced dip in bone marrow EPC numbers is observed. This theory is supported by our data which shows that most circulating endothelial cells also express EPC markers (Figure 5), and thus, are likely to have recently entered the circulation from the bone marrow.

Similar to previous studies of full-length PEDF, the systemic injections of PEDF-34 significantly reduced the progression of retinal neovascularization in the OIR mouse model. Furthermore, PEDF-34 blocked the OIR-induced spike in circulating endothelial cells at P16. This strongly suggests that PEDF-34 inhibits retinal neovascularization by targeting and reducing circulating endothelial cells, although the mechanism by which PEDF-34 reduces circulating endothelial cells and EPCs is unclear. Our finding that PEDF-34 inhibits cell proliferation and induces apoptosis in primary endothelial cell culture suggests PEDF-34 may directly target circulating endothelial cells and EPCs to induce apoptosis or inhibit cell proliferation. Another possibility is that PEDF-34 may block the release of EPCs from the bone marrow into the circulation. Regulation of EPC release is not well understood. However, VEGF is known to play an important role [6]. We measured VEGF levels in the plasma following treatment with PEDF-34 and found that VEGF levels in the plasma were not affected by systemic administration of PEDF-34 (Supplemental Figure  2). However, our previous studies have shown that PEDF competes with VEGF for binding to VEGF receptor 2 (VEGFR2) [24], which accounts for some of the antiangiogenic activity of PEDF. Thus, it is possible that PEDF-34 could bind to VEGFR2 on EPCs and impede VEGF signaling to reduce the VEGF-induced stimulation of EPC release without reducing VEGF levels. Alternatively, PEDF-34 may primarily target the existing vasculature and neovascular lesions to reduce the expression of cell adhesion molecules and soluble signaling molecules, such as *α*_5_ *β*_3_ and *α*_5_ *β*_5_ integrins, intercellular adhesion molecule 1 (ICAM1), and vascular cell adhesion molecule 1 (VCAM1), which act as recruitment signals for circulating endothelial cells and EPCs. In this case, the PEDF-34-mediated reduction in circulating endothelial cells and EPCs could be an indirect result of PEDF-34 targeting the vasculature to decrease expression of recruitment factors.

## 5. Conclusion

This study is the first to demonstrate that an antiangiogenic peptide, PEDF-34, reduces circulating endothelial cells during ischemia-induced neovascularization. This strongly suggests that the PEDF-34 peptide combines antivasculogenic activity and antiangiogenic activity in one peptide. Thus, the PEDF-34 peptide could be a superior biological therapeutic for the treatment of pathological neovascularization, such as proliferative diabetic retinopathy. Since PEDF-34 is a fragment from an endogenous human protein which exists in normal human tissues, and because the small PEDF-34 peptide can be generated in large quantity with high purity by solid phase synthesis, PEDF-34 has great potential for large-scale pharmaceutical development.
